# Podcasting as a novel way to communicate with medical school applicants

**DOI:** 10.1007/s40037-014-0123-2

**Published:** 2014-05-28

**Authors:** Benjamin D. Ferguson, Mary K. Bister, Joni N. Krapec

**Affiliations:** 1 Pritzker School of Medicine, University of Chicago, Chicago, IL USA; 2 Department of Surgery, University of Chicago, 5841 S. Maryland, MC6040, Chicago, IL 60637 USA; 3 Office of Career Advancement, University of Chicago, Chicago, IL USA; 4 Department of Emergency Medicine, University of Cincinnati, Cincinnati, OH USA

**Keywords:** Admissions, Medical education, Podcasting, Recruitment, Social media

## Abstract

Podcasting in medical education is becoming more widely used and may be a useful tool for communicating with applicants to medical school. Given recent trends in the popularity of podcasting and mobile media, we created a podcast to communicate more effectively with applicants to our medical school as well as with the broader premedical community. The purpose of this study was to characterize the listening habits and motivations of our audience and compare the podcast’s benefits to those of other resources. We additionally sought to understand patterns by which our podcast was consumed by a premedical audience. We surveyed medical school applicants who interviewed at the University of Chicago Pritzker School of Medicine for matriculation in 2013. Forty-one percent of those surveyed had listened to the podcast prior to their interview. Only 12 % of listeners accessed the podcast using a mobile device. Ninety-two percent of listeners felt that it faithfully represented the medical school, and 81 % felt that listening would encourage the decision to matriculate. A majority of listeners responded that the podcast was more helpful than other traditional resources. This is the first use of podcasting in medical school admissions and represents a novel way to communicate with prospective students. Our findings demonstrate that podcasting can be an effective tool for communicating with applicants to medical school and highlight its usefulness in recruitment. This method of communication could be adopted by other medical schools to enhance the ways in which they inform their own prospective medical students.

## Introduction

iPod and mp3 player use has grown substantially over the past decade, and as a result, podcasting has emerged as a form of mobile media and distribution of educational, news, and other materials across broad-ranging topics. Podcasting—a portmanteau word combining ‘iPod’ and ‘broadcasting’—involves the dissemination of audio and/or video content freely on the web through the use of Really Simple Syndication, or RSS. This medium has increasingly been utilized by scientific and medical publishers, institutions, and individuals to communicate information to readers, patients, fellow scientists and clinicians, and anyone else who has an interest in their materials.

Podcasts have started to come into use specifically for medical education purposes in recent years. A group of educators at the University of Ottawa has shown their value in teaching anatomy to medical students [1], while another surgeon has used podcasting for educating medical students rotating through the general surgery clerkship at the University of Alberta [2]. This latter resource is also frequently used by an international audience of medical students and residents interested in learning about common topics within surgery [3]. Cheston et al. [4] have assembled an excellent review of the uses of podcasting and related resources in medical education.

In light of these trends, we created a podcast [5, 6] in an effort to communicate more effectively with applicants to our medical school as well as with the broader premedical community. By recording brief, focused audio episodes and posting them publicly on the internet, we have aimed to develop supplementary admissions and institutional information to which applicants would not necessarily otherwise have access using traditional educational materials. To date, there have been over 525,000 unique downloads (including streams) of this resource.

Here, we describe the listening habits and motivations of our audience and explore whether podcasting can be as beneficial as traditional resources, such as websites, email newsletters, blogs and forums, and hard-copy materials, in providing admissions information to medical school applicants.

## Methods

Between August 2012 and March 2013, medical school applicants who interviewed at the University of Chicago Pritzker School of Medicine for the class matriculating in 2013 were emailed a link to a web-based survey about the Pritzker Podcast shortly after their interview. The items included in the survey are provided in the Supplementary Information. Subjective items allowed listeners to select multiple responses as necessary. Items assessing listener benefits employed a three-point Likert scale following a negative-neutral-positive pattern. For other quantitative or comparison items, only a single response was allowed. Link URLs were personalized for each recipient to prevent multiple survey submissions, and responses were anonymized to protect applicant privacy and to encourage honest responses.

## Results

The overall survey response rate was 68 % (412/609). Of the 412 respondents, 41 % (168/412) had listened to our podcast and were therefore queried for the remainder of the survey items. Seventy-four percent (124/168) of listeners discovered our podcast through a direct link on our medical school’s homepage, while 20 % (33/168) and 19 % (32/168) came to it through search engine results and through a post on a Student Doctor Network forum (a popular online pre-health discussion board) thread, respectively, as the next commonest acquisition sources. Eighty-four percent (141/168) of listeners responded that they used our podcast to inform them about features of our medical school, which was the most frequent response; 67 % (112/168) and 63 % (105/168) listened to understand what it would be like to be a medical student at our institution and to inform them about the process of applying specifically to our medical school, respectively.

Despite the popularity of mobile audio devices, 84 % (139/166) of listeners accessed our podcast using a personal computer, while only 11 % (19/166) of listeners used an iPod, iPhone, or other mobile device to access its content. Similarly, only 47 % (78/166) of listeners typically downloaded audio files, with 36 % (59/166) preferring to stream audio only in lieu of downloading.

**Fig. 1:**
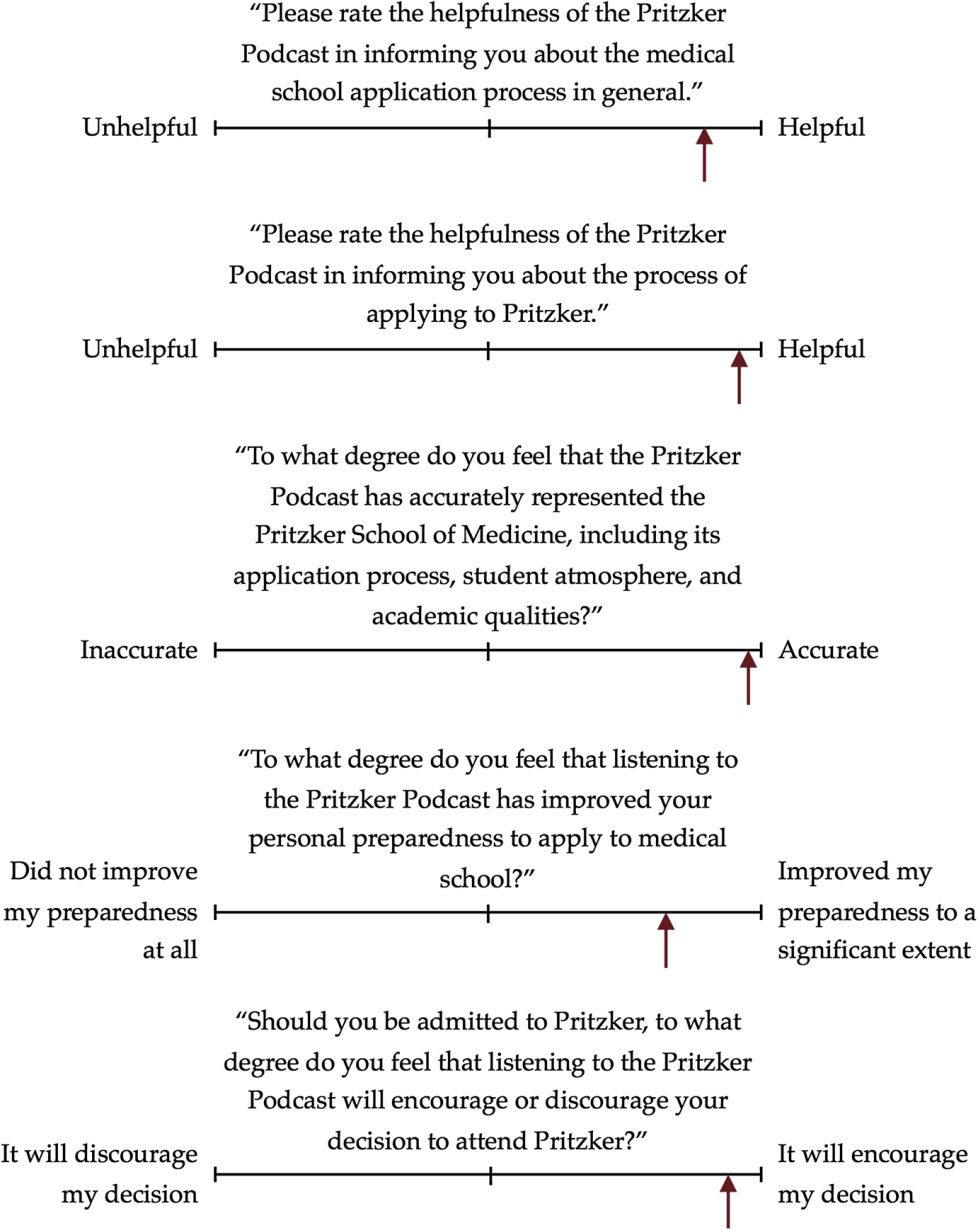
Summary of responses to Likert scale-based survey items. The *arrow* indicates the average listener response along the scale

Seventy-one percent (119/168) of listeners stated that our podcast was helpful in informing them about the general process of applying to medical school, and 87 % (146/168) felt it was helpful for applying to our medical school specifically. Ninety-two percent (152/165) replied that it was an accurate representation of our medical school and its application process, student atmosphere, and academic qualities. Fifty-two percent (86/165) felt that it improved to a significant extent their personal preparedness to apply to medical school, and 81 % (133/164) stated that having listened to our podcast would encourage their decision to attend our medical school should they ultimately be accepted (Fig. 1).

**Fig. 2:**
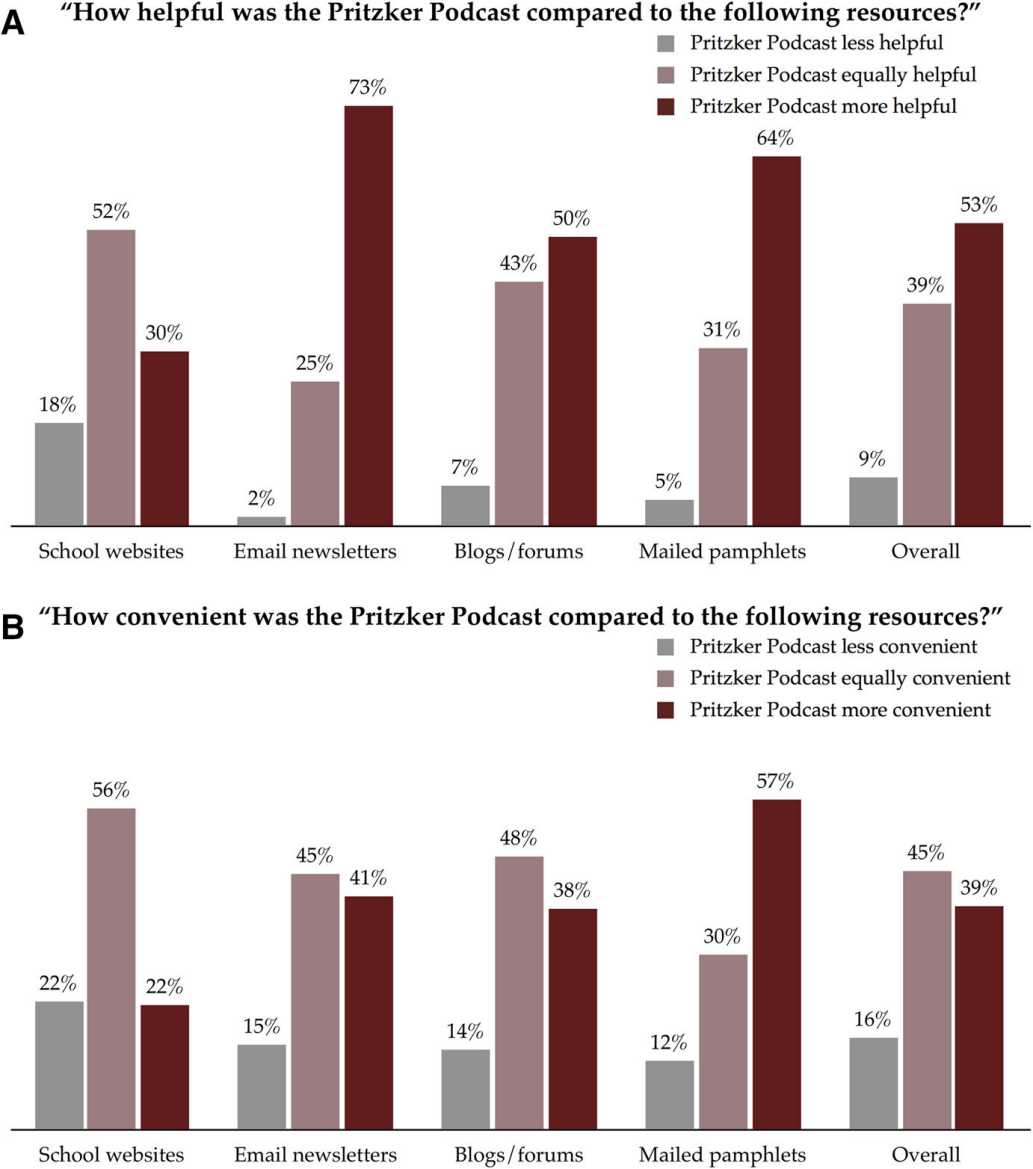
Comparison of the helpfulness and convenience of the podcast with those of conventional information sources. ‘Not applicable’ responses were not included in the analysis

The majority of listeners felt that our podcast was more helpful than email newsletters, blogs and forums, and hard-copy materials, and over half stated that our podcast was as helpful as medical schools’ websites despite the substantial difference in content delivery between these two resources (Fig. 2a). Similarly, the majority of listeners responded that our podcast was equally or more convenient than these traditional resources (Fig. 2b).

## Discussion

Although podcasting has been in existence for over a decade, it has only recently been brought into use by the medical education community. Our survey results demonstrate that podcasting can be an effective tool for communicating with applicants to medical school. Nearly half of our interviewees had listened to our podcast, and the majority did so to learn more about our medical school. Over three-quarters of listeners felt that our podcast would encourage their decision to attend our medical school, highlighting its usefulness from a recruitment perspective. Finally, the vast majority of our audience responded that it is at least as helpful and convenient as conventional premedical communication outlets.

Medical schools’ websites were rated as more helpful and more convenient than our podcast by 18 and 22 % of listeners; this is not an unexpected finding given the detail and dynamic nature of such resources. Additionally, email newsletters, blogs/forums, and mailed pamphlets were rated as more convenient than our podcast by 15, 14, and 12 % of listeners, respectively. These modalities all share with podcasting the benefit of passive communication of information—that is, information is transmitted to its audience without a concerted effort on the listener’s part to obtain it. It is not surprising that some among our audience find consumption of written materials at one’s own pace over listening to audio, which may require a larger and more contiguous time commitment and higher level of attention, and we feel that these data may simply reflect those preferences.

Podcasting on the topic of medical admissions and medical student life has proven to be both easy and extremely inexpensive and has provided myriad advantages over website-, weblog-, and brochure-based complements, including listener convenience, enhanced breadth of focus, increased depth of topical discussion, and improved internet presence, in addition to the empirical data we demonstrate here regarding its effectiveness for educating prospective applicants. The implementation of podcasting in our admissions efforts is consistent with the rapidly growing internet and social media use among young adults over the past several years; [7] this pattern of internet and social media use and awareness has been seen recently within higher education and especially within medical education as well, as implications for online professionalism and its changing definition have come into question [8–15].

To our knowledge, this is the first use of podcasting in medical school admissions and represents a novel way to communicate with medical school applicants and prospective students. We feel that this is a convenient and inexpensive method of medical school–applicant interaction that with time could be adopted by other medical schools to improve or supplement the ways in which they inform their own prospective medical students on school-specific issues and aspects of their application processes, as our listener data indicate that it is both an effective resource for prospective students and potentially an influential tool to be used more directly for recruiting efforts.
